# Latent Insanity

**Published:** 1843-04

**Authors:** 


*Latent Insanity.*—In many cases the experienced eye of the medical practitioner may be able to detect the existence of latent insanity. In such instances the patient evinces no evident indication of any irregularity of mind. His dearest friends and constant companions can perceive no alteration in manner sufficient to excite a suspicion of insanity. Occasionally the patient may manifest a strangeness and oddity of conduct, which is, however, but little noticed. The individual may, notwithstanding, be suffering from incipient symptoms of derangement. He has not altogether lost all power of controlling his feelings or ideas; an internal struggle may be going on between what he knows to be a fact, and the false conceptions which almost like an avalanche are forcing themselves upon his mind. None but those who have gone through this dreadful ordeal can form an accurate notion of the mental agony which a person so unhappily situated experiences! Dr. Darwin relates the case of a most elegant lady who suddenly became melancholy. She retained, however, so great a command over herself, that she was enabled to do the honors of her table with grace and apparent ease. After many days' entreaty, she informed her physician that she thought her marrying her husband had made him unhappy (though it was a love-match on both sides), and that this idea she could not efface from her mind, day or night. It is astonishing for what a length of time a patient under such circumstances will struggle against a delusion which is endeavoring to fasten itself upon the mind. In such cases the patient gives no marked indication of mental unsoundness; she will attend to domestic duties, mix as usual with parties of pleasure, take her accustomed seat at the opera, and yet manifest no sign of the tempest raging within. How often do such patients confess to the medical practitioner, when the state of mind becomes so apparent as to compel the friends to summon relief, that for months they have been contending courageously against what they were conscious were erroneous perceptions. Hoping eventually to master these false ideas, they have concealed, from those in whom confidence ought to have been placed, their melancholy condition. This state of mind is fraught with much cerebral mischief. It cannot exist for any length of time without serious consequences ensuing.

*F. Winslow's Health of Body and Mind.*

